# PD72 Are Patient Experience Data Currently Embedded In Reimbursement Decision-Making? An Analysis Of Belgian Reimbursement Documents

**DOI:** 10.1017/S0266462324003295

**Published:** 2025-01-07

**Authors:** Alice Vanneste, Charlotte Verbeke, Katrien Deschepper, Danique Donvil, Liese Barbier, Rosanne Janssens, Isabelle Huys

## Abstract

**Introduction:**

Stakeholders in the drug life cycle recognize the importance of integrating patient experience data (PED) into healthcare decision-making. PED includes patient input, patient reported outcomes (PROs), patient reported experiences, and patient preference information. However, it remains unclear if, how, and to what extent PED are used in reimbursement decisions in Belgium.

**Methods:**

A document analysis was performed to evaluate and compare the reporting of PED in Belgian reimbursement documents for COVID-19, oncology, and cardiology. Documents of medicinal products that received a European marketing authorization between 1 January 2015 and 30 September 2023 and were reimbursed in Belgium were included. Data were analyzed descriptively and qualitatively.

**Results:**

Preliminary results showed that PED was generally either not reported or not explicitly reported in Belgian reimbursement documents. In some documents, patient reported outcome measures (PROMs) were mentioned. Additionally, few documents stated PROs such as quality of life. PROMs, PROs, and quality of life outcomes appeared in the reimbursement dossier section describing the therapeutic value of the product. From the preliminary findings it seems that PED were more often present in oncology documents. The reimbursement dossiers did not report whether or how PED were used to inform a particular reimbursement decision.

**Conclusions:**

Preliminary findings suggest that PED are not systematically included in Belgian reimbursement documents. To improve transparent submission and reporting of PED in reimbursement dossiers, guidance from reimbursement agencies as well as checklists and evaluation templates are needed.

